# Lateral transport osteogenesis in maxillofacial oncology patients for rehabilitation with dental implants: a retrospective case series

**DOI:** 10.4317/medoral.18103

**Published:** 2012-12-16

**Authors:** Arturo Bilbao-Alonso, José M. García-Rielo, Pablo Varela-Centelles, Juan Seoane

**Affiliations:** 1 Service of Oral and Maxillofacial Surgery. Santiago de Compostela University Hospital. Santiago de Compostela (A Coruña). Spain; 2 Stomatology Department. School of Medicine and Dentistry. University of Santiago de Compostela. Santiago de Compostela (A Coruña). Spain

## Abstract

Objectives: To report on the use of lateral transport osteogenesis in cancer patients after maxillo/mandibular resections and on the implant survival rate in the generated bone Material and Methods: Four patients treated using lateral transport osteogenesis entered this descriptive study and were retrospectively studied (mean age 55; range 41-62). Results: Reconstruction of segmentary defects after surgical and radiological cancer treatment on maxilla and mandible was achieved. No relevant intra- or post-operative complications occurred. No differences on implant survival were observed between patients who had received radiotherapy and those who had not. Conclusions: This approach can be considered a recommendable reconstructive option after oral cancer treatment –including radiotherapy- particularly for high-surgical-risk, collaborative patients.

** Key words:**Distraction osteogenesis, oral cancer, radiotherapy, reconstruction, dental implants.

## Introduction

Many maxillofacial tumours are diagnosed at advanced stages with frequent mandible/maxillary involvement, resulting in marginal or segmental resection with adjuvant radiotherapy. The vascularised free-osseous flap (VFOF) is the current gold standard for reconstruction in these situations (1) although this procedure is far from ideal for patients with increased surgical risk and for those requiring an adequate soft tissue quality before implant insertion, as VFOF results in a too thick overlying soft tissue without peri-implant attached gingival (1,2).

Osteogenic distraction procedures, like transport-disc-distraction-osteogenesis (TDDO), may solve these shortcomings as no statistically significant differences could be found between autogenous bone and distracted bone sites in terms of stability and im-plant survival rate (3-6). The main limitation of such techniques in these situations would come from the effects of radiotherapy, applied either before or after distraction, on the regenerated bone (7,8).

This paper reports on the use of lateral transport osteogenesis in cancer patients who have undergone maxillo/mandibular resections and on the implant survival rate in the generated bone.

## Material and Methods

**Figure 1:**
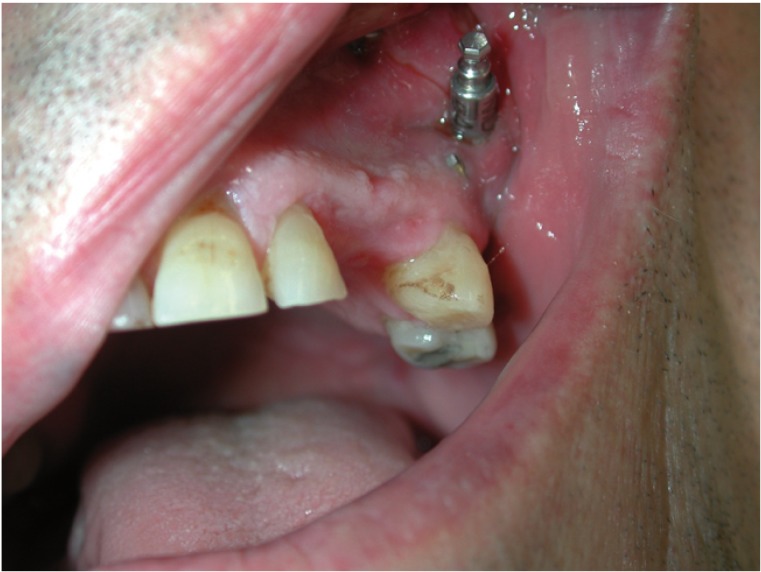
Patient number 3 after lateral transport osteogenesis with adequate gingival tissues for implant placement.

**Figure 2:**
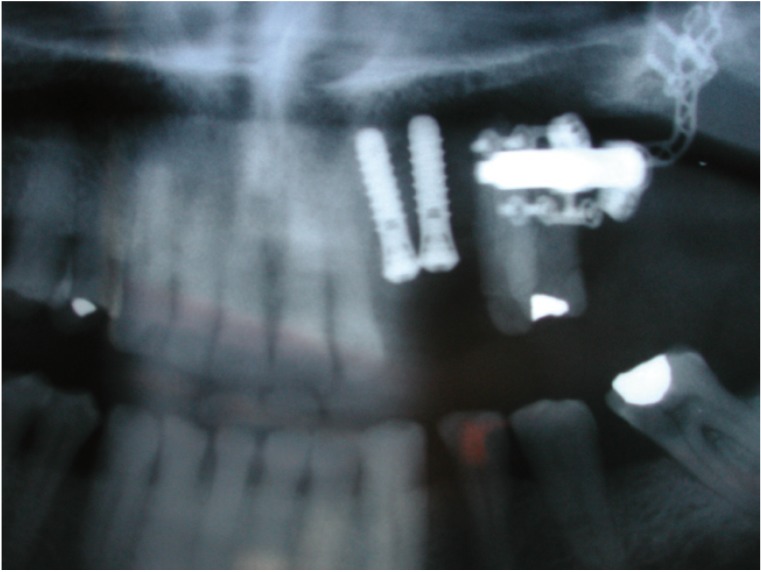
Detail from orthopantomograph image showing implants placed in newly generated bone before osteodistractor removal.

Four patients with segmental defects after oncological resection were descriptively studied (mean age 55; range 41 to 62) ( Table 1). The lateral bone transport technique was used to reconstruct the maxillary and mandibular bone defects in all cases: periosteum over the designed transport disk was preserved during the procedure to ensure vascularity, and the ostectomy was performed by means of a piezoelectric device (Piezosurgery System; Mectron Medical Technology, Carasco, Genoa, Italy) 7 to 15 mm away from the defect to create the transport disk. The MODUS modular distractor (Medaris AG, Basel, Switzerland) was used in the maxilla and the KLS system (Martin intraoral distractor, USA) in the mandible (Figs. 1,2). The devices were not activated until the 5th or 6th day (latency period). The chosen distraction/contraction protocol progressed at a distraction rate of 0.75 mm a day for three days to contract another 0.75 mm on the forth day -in order to avoid excessive tension on the soft tissues- and continued until the device’s distal stump was reached. A consolidation period of 8 to 12 weeks was allowed, and the dental implants were placed (4.1 mm diameter, 12 mm long, Standard Plus, with a SLA® surface Straumann AG, Waldenburg, Switzerland ). Patients were followed for periods ranging from 3 to 9 years, under a protocol that included clinical and radiographic assessment with intraoral radiographs at the time of implant placement and at 1, 3, 6 and 12 months and yearly thereafter.

**Table 1:**
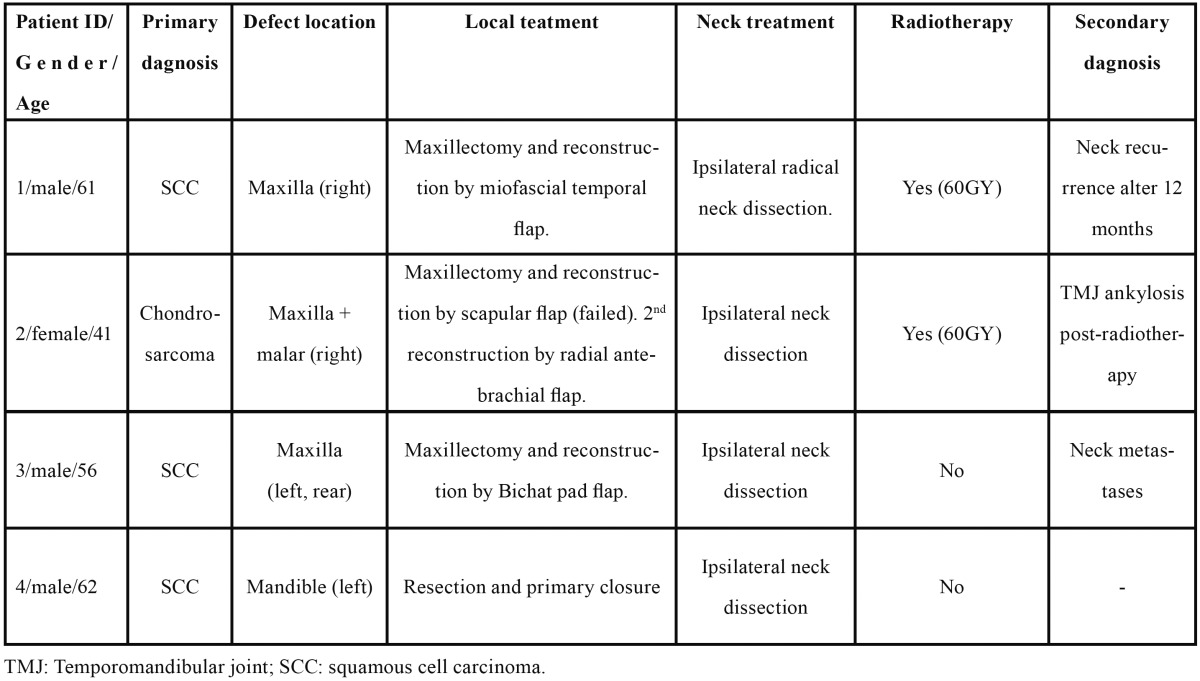
Patients’ clinical and pathological features.

The variables considered in the study included an assessment of surgical intra and postoperative morbidity, defect location and size, transport disc length, length of the distracted bone, consolidation period and survival rates.

## Results

The results are summarized in  Table 2. The use of lateral transport osteogenesis techniques in these series has permitted the reconstruction of segmentary defects between 30 to 55 mm length, after surgical and radiological maxillofacial cancer treatment. None of the cases showed relevant intra- or postoperative complications. No differences in terms of implant survival were observed between the two patients who had received radiotherapy and those who had not. In both situations, a 100% implant survival rates could be achieved for the implants placed in TDDD generated bone.

**Table 2:**
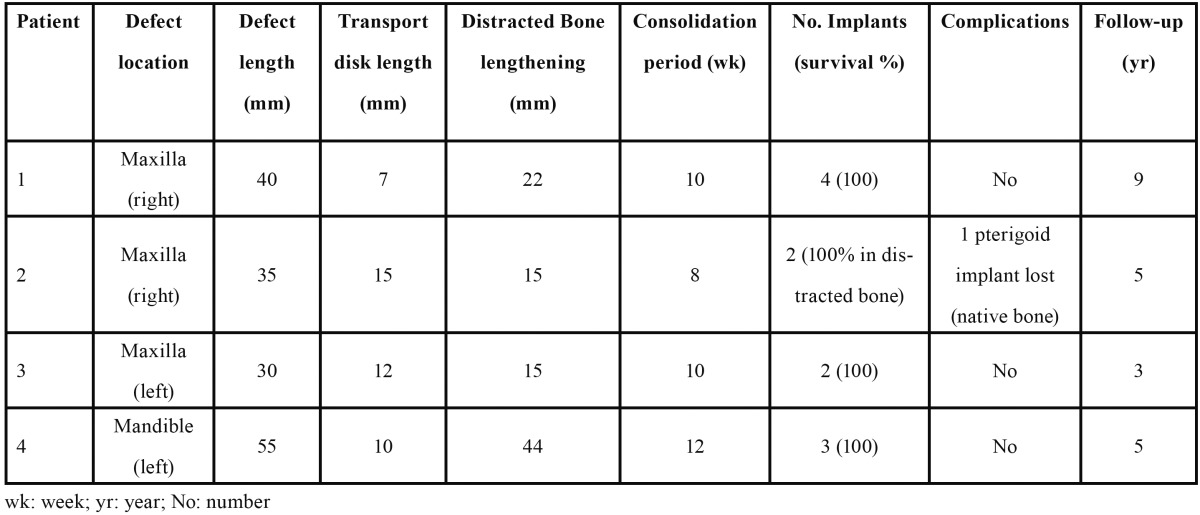
Lateral transport osteogenesis description.

## Discussion

TDDO has been recently recognised as a valuable alternative for mandibular reconstruction after surgical resection and radiotherapy, producing functional bone similar to residual bone (3,8). Short case series, mostly reporting on mandible, show success rates close to 83% (5), but the information available about this procedure for maxillary reconstruction and the influence of radiotherapy on its results is limited. This may well be due to the difficulty to obtain an adequate morphology on curved segments, which could make a 2-phase distraction mandatory. Moreover, implants placed in maxilla after radiotherapy have proved a poor survival rate (59%), (9) although studies on this situation are so scarce that no definitive conclusions can be drawn.

In this series, all 6 implants inserted in patients who had received maxillary radiotherapy (patients 1 & 2) elicited a 100% five-year survival rate, despite the fact that the described procedures are different from more conservative protocols (0.5 mm a day and/or twice long consolidation period) reported in the literature (10).

It is concluded that, with the inherent limitations to such a small case series, TDDO may well be considered a recommendable reconstructive option, both for maxilla and mandible, in patients having undergone oral cancer therapy –including radiotherapy- particularly for high-surgical-risk, collaborative patients. However, controlled randomized clinical trials supporting this therapeutic approach are needed to endorse this technique.
